# Sirenomelia (Mermaid Syndrome): A Case Report*

**DOI:** 10.5146/tjpath.2020.01491

**Published:** 2020-09-15

**Authors:** Şirin Küçük, İzzet Göker Küçük

**Affiliations:** Department of Pathology, Faculty of Medicine, Usak University, Uşak, Turkey; Spec. Dr., Kemal Öz Family Health Center, Uşak, Turkey

**Keywords:** Sirenomelia, Fusion of lower extremities, Caudal regression syndrome

## Abstract

Sirenomelia, which is also known as mermaid syndrome and characterized by the fusion of the lower extremities, is the most severe form of caudal regression syndrome and one of the rare and lethal congenital malformations. The anomalies that might be seen in this syndrome include pelvic-sacral dysplasia, genital anomalies, bilateral pelvic renal fusion accompanied by renal dysplasia, colon atresia, unilateral umbilical artery, and imperforated anus. The incidence of sirenomelia is 0.8-1 cases in 60,000-100,000 deliveries and the male/female ratio is 2.7-3:1. The case reported in the present study was a 13-week-old male fetus 30 g in weight with a macerated appearance. The upper extremities had a relatively normal appearance but the lower extremities were conjoined and there was a single lower extremity consisting of conjoined feet and toes. In the face, the nasal bridge was sunken, the ears had a low position, and there were cleft palate and cleft lip. Examination of the external genital organs revealed that the penile part was in the anal region. There was no anus opening. The crown-rump length was 8.5cm, the heel-toe length was approx. 1cm, and the rump-heel length was approx. 3.7cm. There were none of the two kidneys, ureter, bladder, urethra, or rectum. In the umbilical cord, there were 2 venous structures, one of which was the artery. Perivillous congestion and hyperemia, perivillous calcification, deciduitis, and focal infarct regions were observed in placental tissues. This report aims to discuss this very rare case together with the literature.

## INTRODUCTION

Sirenomelia is a rarely seen and fatal disease with severe malformation in lower extremities, in which the legs are attached to each other and there are various anomalies related to the visceral organs. Some of the most frequently affected organs is the genitourinary system and the gastrointestinal system (1–21). Its worldwide incidence ranges between 1.1 and 4.2 per 60,000-100,000 deliveries (1). These fetuses generally die either during the pregnancy or immediately after the delivery. The syndrome is more frequently seen in one of the monozygotic twins (3,18). Although its etiology is not exactly known, maternal diabetes, genetic susceptibility, and vascular hyper-perfusion are considered among the causes. Although a possible mechanism is that the extremity bud cannot separate from the primordial cells due to the lack of the posterior mesoderm axis and is unable to rotate during the organogenesis period, it has then been shown that the defect develops in the early period of vascular development. Two main causes were claimed for its pathology: a developmental anomaly in the veins feeding the lower extremities and an anomaly in mesodermal cell migration (1,3,10–12,14,16–19). Based on the type of fusion, sirenomelia is classified as sympus dipus or symmelia, sympus monopus or uromelia, and sympus apus or sirenomelia (1–5). In the literature, surviving cases have been reported very rarely (2,11). The present case is discussed together with the literature since the condition is rare.

## CASE REPORT

**Figure 1:**
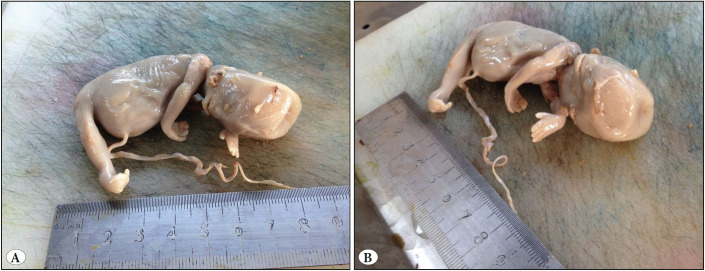
Sirenomelic fetus having lower extremities attached as single extremity, fused fingers and feet, flat nasal base, low-set ear, cleft palate, and cleft lip.

**Figure 2:**
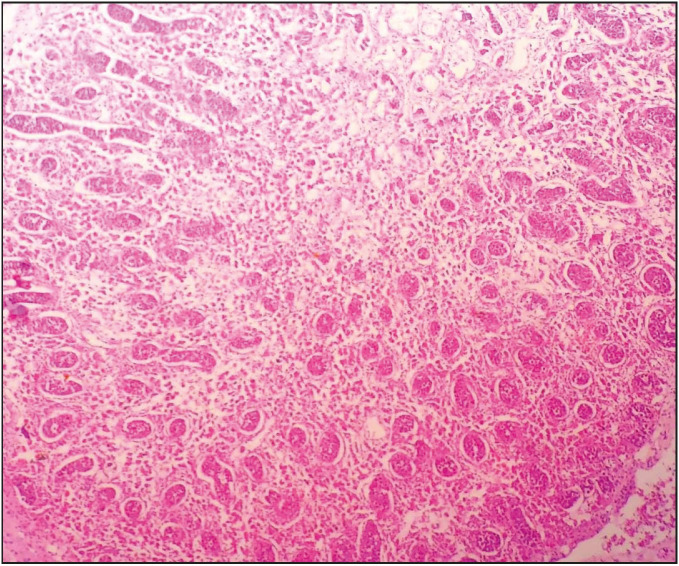
Immature, desquamated and necrotic testis tissue (H&E; x40).

**Figure 3:**
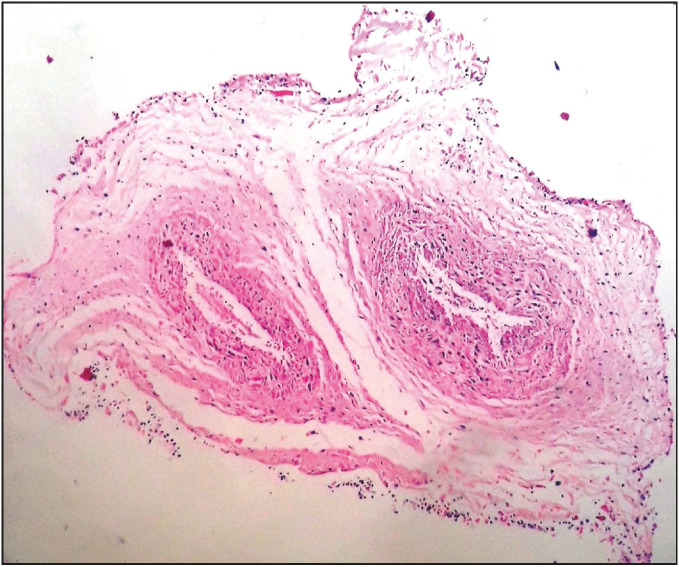
Umbilical cord consisting of two venous structures, an artery and a vein (H&E; x20)

A 30-year-old pregnant women at the 13th gestational week (G2, P1, A0, L1) presented at the emergency service with complaints of vaginal bleeding, inguinal pain, and nausea. On physical examination, vaginal bleeding and sensitivity at the suprapubic region were detected. The other examination findings were normal and there was nothing characteristic in the patient’s history. Obstetric ultrasonography (USG) was performed. CRL was found to be 60 mm, which is compatible with 12 weeks and 3 days. There was no cardiac or fetal activity, the amniotic fluid was significantly decreased, and the internal os was closed. No characteristic findings were observed in whole blood, biochemistry, hormone, TORCH, and ELISA tests. Among the coagulation tests, aPTT was found to be slightly prolonged. The blood type was 0 Rh (+). Based on these findings, the patient was diagnosed with in utero mort fetalis (I.U.M.F.) and hospitalized at the obstetrics and gynecology clinic. Revision-curettage (R/C) was performed. The curetted material was sent to pathology. No bleeding or complication was observed during the follow-up period. In the macroscopic examination, the fetus was found to have a completely macerated appearance, 30 g of weight, a hydropathic head, umbilical cord rotating around the neck several times, relatively normal upper extremities, conjoined lower extremities, and a single lower extremity with conjoined toes and feet. Examining the external genital organs, it was observed that the penis was in the anal region. There was no anus opening (imperforated anus). In the face, the nasal bridge was sunken and the ears were low in position, besides the cleft palate and cleft lip (Figure 1). The crown-rump length was measured as 8.5 cm, the heel-toe length approx. 1 cm, and the rump-heel length approx. 3.7 cm. There were none of two kidneys, ureter, bladder, urethra, or rectum. There was a single testis (Figure 2). The organs were found to be immature. There were several hemorrhagic foci on the placenta. On microscopic examination, necrosis and widespread cytolysis were observed in the liver, intestine, bladder, the single testicle, and the heart in identifiable regions. There were two venous structures in the umbilical cord. One of them was the artery and the other one was the vein (Figure 3). Perivillous congestion and hyperemia, perivillous calcification, deciduitis, and focal infarct regions were observed in the placental tissues. According to POTTER’s 2007, the fetus was found to correspond to the 12th-13th gestational week. Based on the microscopic and macroscopic findings, the case was diagnosed as sirenomelia.

The informed consent form was obtained from parents.

## DISCUSSION

Sirenomelia is a rare congenital malformation, which is seen in approx. 1/60,000-1,000,000 deliveries (1–19). The number of cases reported to date is approx. 300 (18,21). According to the results obtained from the studies carried out on mice and aiming to discover how this defect develops (the genetic basis), it was claimed that this defect develops as a result of excessive secretion of retinoic acid (RA) (disruption of Cyp26a1 enzyme causing a gain of RA function) and the lack of signalization of bone morphogenetic protein (Bmp) to the lower part of the body among humans. Reduction of Bmp signalling in the ventral caudal embryonic mesoderm leads to the occurrence of several caudal defects, severe cardiac malformations and absence of major vessels. RA and Bmp thus regulate each other. The genetic reasons also include balanced translocation including chromosome 16 and triploid mosaic (16–21). In our case, no genetic analysis was performed. There are two common hypotheses for sirenomelia. According to the first hypothesis, the lack of blood supply in the lower extremities develops as a result of the abdominal and umbilical venous structure. According to the second hypothesis, a lack of caudal mesoderm develops as a result of a defect in blastogenesis and the malformations in the lower portion of body develop due to a caudal embryologic development disorder in the early gestation (1,6,7,10–12,14,16–21).

Several risk factors related with the sirenomelia malformation are maternal diabetes, retinoic acid, exposure to heavy metals and teratogens, genetic factors, monozygotic twins, male gender, and mother’s age <20 years or >40 years (1,3–12,18). There is a strong relationship between sirenomelia and maternal diabetes; the relative risk ratio is 1/200-250, and this ratio was found to be 22% among the fetuses with anomaly (8,10,11,16–18). The male/female ratio is 2.7-3 (5,6,16–18). In the present case, none of the known risk factors except for male gender was detected.

The most characteristic finding of sirenomelia is the fusion of lower extremities (1–14,18). The disease has a wide diversity ranging from the mildest form that is fusion, in which all the lower extremity bones exist, to the most severe type, in which there is only rudimentary bone. Significant efforts have been made in order to classify sirenomelia. However, the classification that is most widely used nowadays is the one developed by Stocker and Heifetz, which has been used since 1987 (Table 1). This classification was developed based on the presence of the skeletal elements in the thigh and leg. In Type I, which is the mildest form, all the bones are present and fusion is observed only in superficial tissues. In Type VII, which is the most severe form, there is a single bone and the legs and feet are not present (1,7,8). Although there was no radiological finding available, the present case was found to correspond to Type IV or sympos monopus in terms of macroscopic appearance.

**Table 1:** Classification of Sirenomelia by Stocker and Heifetz

**Type**	**Characteristics**
I	All thigh and leg bones present
II	Single fibula
III	Absent fibula
IV	Partially fused femurs, fused fibula
V	Partially fused femurs, absent fibula
VI	Single femur, single tibia
VII	Single femur, absent tibia

The visceral organ malformations seen in sirenomelia include various grades of renal and urethral dysplasia. Absence of one or both kidneys (frequently the total renal agenesis), cystic malformations in kidneys, absence of the bladder, and urethral atresia can be seen (1,6–8,12,13,16,18). In addition to the renal agenesis, the present case also had absence of the ureter, urethra, and rectum. Ectopic renal tissues may be seen at various points of pelvis because of the anomalies in the migration of metanephritic tissue. The most important genital anomalies are the external genital organ malformations. Either the external genital structures are absent or they are located at a different position and the gonads are generally not affected. In the present case, the penis was in the anal region and not at its normal position. However, contrary to the literature, there was a single testis. Gastrointestinal anomalies are frequently seen and some of these are the blind-ended colon, rectal atresia, and imperforate anus (1,5–7,12,18). An imperforate anus was observed in the present case. The venous malformations seen in this disorder include a dual venous structure (an umbilical artery and an umbilical vein) instead of a triple venous structure (two umbilical arteries and an umbilical vein) (1,5–8,10,12,13,18). In the present case, there was a dual venous structure consisting of an umbilical artery and an umbilical vein.

The differential diagnosis includes bilateral renal agenesis, kidney malformations, single umbilical artery, megacystis, VACTERL/VATER (vertebral defects, anal atresia, cardiac defects, tracheo-esophageal fistula, renal anomalies and limb abnormalities) association, and caudal regression syndrome (CRS). Although sirenomelia may be regarded as a severe form of the latter, the two entities represent distinct nosologic conditions and should receive individual genetic counseling (21).

CRS is a broad term that refers to a heterogenous constellation of congenital caudal anomalies affecting the caudal spine and spinal cord, the hindgut, the urogenital system, and the lower limbs (16,17,19–21). In fact, while CRS is hypothesized to arise from a primary defect of the caudal mesoderm that interferes with the formation of the notochord, the primary mechanism leading to sirenomelia is the diversion of blood flow away from the caudal portion of the embryo through a single vitelline artery (21). As a result, fetuses with caudal regression show two umbilical arteries, two hypoplastic lower extremities, and non-fatal kidney findings with imperforate or normal anus, while sirenomelic fetuses have renal agenesis or dysgenesis, a single aberrant umbilical artery, fused sub-extremities, and imperforate anus (20,21). We diagnosed our patient with sirenomelia due to the presence of single umbilical artery, a single testis, imperforate anus, fused lower extremities, cleft palate, cleft lip, total renal agenesis, and absence of the bladder, ureter, urethra and rectum.

The other anomalies that might be seen in sirenomelia include sacral agenesis, absence of gallbladder and spleen, omphalocele, lordosis, malformed vertebra-hemivertebra, and central nervous system (CNS) anomalies, as well as less frequently observed ones such as cleft palate, upper thoracic and cervical vertebra anomalies, pulmonary hypoplasia, and cardiac anomalies (1,6–8,12,13,16–19). In the literature, adrenomegaly has been detected in the case of a baby with a diabetic mother (15). In the present case, there were sunken nasal bridge, fallen ears, cleft palate, and cleft lip. Ultrasonography (USG) performed in the antenatal period (end of the first trimester) is very important in the diagnosis of sirenomelia. If possible, 3D USG should be preferred over 2D USG. This is because the skeletal anomalies can be better detected using 3D USG (3,13). The absence of fetus movements; adorsal, lumbal, or sacral vertebral gap; short craniocaudal length, and lower extremities with an abnormal appearance can be detected with USG. Oligohydramnios is the finding that is easiest to detect (3,16). In the present case, the pregnant woman was not followed-up on a regular basis and she applied to the emergency unit with complaints of vaginal bleeding, inguinal pain, and nausea. No cardiac or fetal activity was observed, the amniotic fluid volume was significantly low (oligohydramnios), and the internal os was closed. Revision curettage was performed. It was therefore not possible to make an early diagnosis using USG images and the diagnosis was made based on the pathological examination.

In surviving sirenomelia cases, treatment can be provided with a multidisciplinary approach. The conjoined legs can be surgically separated. During the preparation for surgery, subcutaneous tissue expanders such as balloons can be placed and the skin stretched and prepared for enlargement by filling the balloons with saltwater. The enlarged skin is used during surgical separation (6,8,11).

In conclusion, the diagnosis of sirenomelia can be made using ultrasonography performed at the end of the first trimester. Among the ultrasonographic findings, oligohydramnios and lower extremity defects can be useful in the diagnosis. The pregnancy may be terminated in cases of a fetus with a severe anomaly that is inconsistent with survival.

## Conflict of Interest

The authors report no conflicts of interest.
